# A Novel Technique for Identifying the Instar of Field-Collected Insect Larvae

**DOI:** 10.1371/journal.pone.0057836

**Published:** 2013-02-28

**Authors:** Kôji Sasakawa

**Affiliations:** Department of General Systems Studies, Graduate School of Arts and Sciences, The University of Tokyo, Tokyo, Japan; Biodiversity Insitute of Ontario - University of Guelph, Canada

## Abstract

Many field studies of insects have focused on the adult stage alone, likely because immature stages are unknown in most insect species. Molecular species identification (e.g., DNA barcoding) has helped ascertain the immature stages of many insects, but larval developmental stages (instars) cannot be identified. The identification of the growth stages of collected individuals is indispensable from both ecological and taxonomic perspectives. Using a larval–adult body size relationship across species, I present a novel technique for identifying the instar of field-collected insect larvae that are identified by molecular species identification technique. This method is based on the assumption that classification functions derived from discriminant analyses, performed with larval instar as a response variable and adult and larval body sizes as explanatory variables, can be used to determine the instar of a given larval specimen that was not included in the original data set, even at the species level. This size relationship has been demonstrated in larval instars for many insects (Dyar’s rule), but no attempt has been made to include the adult stage. Analysis of a test data set derived from the beetle family Carabidae (Coleoptera) showed that classification functions obtained from data sets derived from related species had a correct classification rate of 81–100%. Given that no reliable method has been established to identify the instar of field-collected insect larvae, these values may have sufficient accuracy as an analytical method for field-collected samples. The chief advantage of this technique is that the instar can be identified even when only one specimen is available per species if classification functions are determined for groups to which the focal species belongs. Similar classification functions should be created for other insect groups. By using those functions together with molecular species identification, future studies could include larval stages as well as adults.

## Introduction

Insects are a group that includes numerous species, but the larval stages are unknown in most species. This trend is particularly strong in holometabolous species, in which larvae and adults have markedly different morphologies [1]. Although it has received little attention, knowledge of larval stages is important in many respects. From an ecological point of view, because the number of larvae exceeds the number of adults, examinations of larval stages are indispensable for estimating the functional role of a species in an ecosystem [2]–[4]. From a taxonomic point of view, larval morphology provides valuable phylogenetic information that cannot be obtained from adult morphology [5]–[7].

The first step in studying insect larvae is to establish a method for species identification. Three primary methods have been used to date, but each has methodological limitations [8]. The first is to identify field-collected larvae using reliable identification keys from neighboring areas (determinatio ex systemate). However, using this method, larvae can be misidentified, and the larval instar cannot be determined [9], [10]. In the second method, species identification is performed by rearing field-collected larvae until they become adults, and the larval description is detailed based on photographs, exuvia(e), and/or individuals that were not reared for species-identification (det. ex evolutione imaginis). However, detailed descriptions based on photographs and exuvia(e) are generally difficult. Additionally, when different individuals are used for species identification and description, misidentification can occur due to confusion between related syntopic species. The third method involves preparing larval specimens of all instars from eggs obtained from adults (det. ex ovipositione). This method is reliable but often labor intensive. Moreover, it cannot be applied to species for which rearing procedures are not established [11].

Recently, a novel method has become available for identifying the species of insect larvae. This method compares DNA sequences from field-collected, unidentified larval samples with those of identified adult samples. In principle, it can correctly identify all larval specimens to species (when particular sequences are used, this method is called “DNA barcoding”; [12]). Using this molecular species identification technique, studies have revealed previously unknown morphological and ecological traits of many species during larval stages [13]–[15]. However, molecular species identification has several methodological limitations [16]. One is the inability to identify the developmental stage (instar) of larvae. From an ecological perspective, identifying the growth stage of each collected individual is indispensable for determining population dynamics in focal species [1]. From a taxonomic perspective, because morphology generally differs among instars in insect larvae (e.g., chaetotaxy), the identification of larval instars is necessary for comparing homological characters among species [17].

Here, using a larval-adult body size relationship across species, I present a novel technique for identifying the instars of field-collected insect larvae that have been identified by molecular species identification technique. This method is based on the assumption that classification functions derived from discriminant analyses, performed with larval instar as a response variable and adult and larval body sizes as explanatory variables, can be used to identify the instar of a given larval specimen that was not included in the original data set. I applied this technique to a data set derived from the beetle family Carabidae (Coleoptera), for which information on adult and larval morphologies is available in many species, and examined the method’s utility and factors that affected discrimination accuracy.

## Materials and Methods

### Key Idea

**Figure 1:**
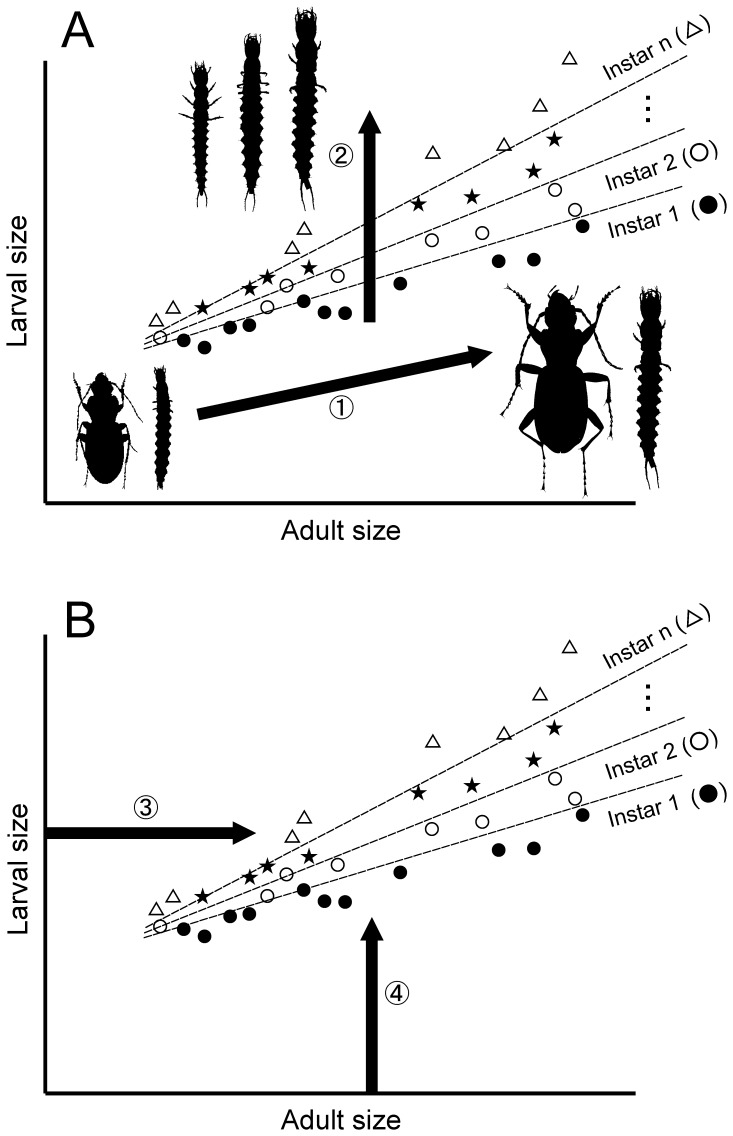
Schematic diagram showing analyses of species with *n* larval instars. On the adult size–larval size plane, “areas” of larval instars are not expected to overlap with each other (A) because among species, species with larger adult sizes have larger larval sizes (arrow 1), and within species, older larvae have larger body sizes (arrow 2). Using this adult size–larval size relationship (B), the instar of a larval sample that was not included in the data set can be identified based on larval size (measured from the specimen; arrow 3) and expected adult size (obtained from conspecific adult specimens or the literature) after molecular methods are used to identify the species of the larval sample; arrow 4). Dotted lines indicate boundaries between instar areas. Note that this diagram is conceptual; in practical analyses (discriminant analyses), the “area” of each larval instar cannot be represented two-dimensionally.

Among related insect species, species with a larger adult body size are expected to have a larger larval body size in a given instar (Arrow 1: Fig. 1A). In each species, older larvae must have larger body sizes (Arrow 2: Fig. 1A). Thus, in a data set composed of related species, one assumes that “areas” for each larval instar on an adult size–larval size plane do not overlap with each other (Fig. 1A). Also, in using this adult size–larval size relationship, one also expects that the instar of a larval sample that was not included in the original data set can be identified based on information about larval and adult sizes (Fig. 1B). Information on larval size can be obtained by measuring the larval specimen. Information on adult size can be obtained through molecular species identification and subsequent measurements of conspecific adult specimens or by using published values. Consequently, the instar of a larval sample can be identified if samples are available for morphological measurements and molecular analyses. Here, this method of identifying larval instar using molecular species identification and morphometric analysis was performed via discriminant analyses.

### Test Data Set

The test data set used in this study was derived from published information on the beetle family Carabidae (Coleoptera). This taxon is one of the most widespread insect groups in the world and includes more than 40,000 described species [18]. Information on adult morphology, including body size, is available for many species, and larval morphology has been described for about 800 species [19], [20]. In Carabidae, larval morphology has often been described based on field-collected specimens that were identified using indirect evidence, such as syntopic occurrence with adults or the consistency of morphology between larvae of related species (i.e., ex systemate; see Introduction). In this study, these results were not used. Only data for which both the species and the instar of described samples were identified without question were used; i.e., larvae reared from eggs obtained from adults (ex ovipositione) or exuvia(e) obtained from rearing field-collected larvae to adults (ex evolutione imagines). In some cases, in addition to these materials, field-collected larvae were used. For species with only two larval instars, such as *Brachinus*, information on the later instar was not used. Consequently, a total of 971 data entries from 399 species were available. All of the data entries included the larval instar (categorical variable with three levels: first, second, or third instar), the larval head width (quantitative variable), adult body length (quantitative variable), and “taxa” (categorical variable; only monophyletic clades supported by molecular phylogenies with high nodal confidence were used [21]–[25]). Additionally, 403 data entries included an additional variable, larval cerci length (quantitative variable). For analyses, two types of data sets were used: data set 1, composed of data entries with larval instar, larval head width, adult body length, and taxa; and data set 2, composed of data entries with these four variables plus larval cerci length. Details of the analyzed data and their references are listed in Table S1 in the Supporting Information.

### Data Analysis

Discriminant analysis was performed with larval instar as a response variable and the other quantitative variables as explanatory variables. The analysis was performed using data sets for overall Carabidae and eight within-family taxa that had more than 10 data entries for each larval instar in data set 1, namely Trechinae, Pterostichini, Zabrini, Harpalini, Sphodrini, Platynini, Chlaeniini+Panagaeini+Oodini, and Lebiini+Cyclosomini. Linear and Mahalanobis squared distance-based discriminant analyses were used (hereafter, referred as LDA and MDA, respectively), but LDA requires homogeneity of the variance–covariance matrices of the data sets. Box’s M tests showed that without transforming the variables, homogeneity could not be assumed for all of the data sets. When the quantitative variables were log(*x*+1) transformed, homogeneity was confirmed in some data sets, but it still could not be confirmed in others (Table 1). However, Box's M test is known to be extremely sensitive, and even very small *p*-values can erroneously suggest heterogeneity [26]. Thus, after the variables were log(*x*+1) transformed, LDA was performed using all of the data sets. MDA could not be performed using data set 2 entries for two taxa (Zabrini and Platynini) because the Mahalanobis squared distance could not be calculated, probably due to multicollinearity between variables.

**Table 1:** Correct classification rates (%) of linear (LDA) and Mahalanobis squared distance-based (MDA) discriminant analyses for various Carabidae taxa.

Taxa^a^	*n* (L1/L2/L3)^b^	LDA				MDA			
		L1	L2	L3	all	L1	L2	L3	all
Data set 1									
Overall Carabidae	358/302/311	70.9	60.3	81.4	71.0	78.2	52.3	80.7	71.0
Trechinae	46/37/31	82.6	89.2	77.4	83.3	89.1	86.5	87.1	87.7
Pterostichini	93/88/87	86.0	84.1	90.8	86.9	88.2	83.0	90.8	87.3
Zabrini*	31/26/27	74.2	69.2	88.9	77.4	87.1	61.5	88.9	79.8
Harpalini	81/70/72	77.8	61.4	80.6	73.5	79.0	58.6	79.2	72.6
Sphodrini*	16/16/17	93.8	93.8	88.2	91.8	93.8	93.8	88.2	91.8
Platynini*	12/11/14	91.7	90.9	100.0	94.6	100.0	81.8	100.0	94.6
Chlaeniini+Panagaeini+Oodini*	15/14/16	93.3	85.7	100.0	93.3	93.3	85.7	100.0	93.3
Lebiini+Cyclosomini*	20/18/18	95.0	83.3	100.0	92.9	95.0	88.9	100.0	94.6
Data set 2									
Overall Carabidae	173/94/136	72.3	56.4	83.1	72.2	76.3	56.4	81.6	73.4
Trechinae	13/9/11	92.3	100.0	81.8	90.9	100.0	88.9	100.0	97.0
Pterostichini*	33/23/26	84.8	78.3	84.6	82.9	90.9	65.2	92.3	84.1
Zabrini	16/3/13	75.0	66.7	92.3	81.2	―	―	―	―
Harpalini	45/24/41	80.0	83.3	87.8	83.6	86.7	87.5	90.2	88.2
Sphodrini*	8/8/7	100.0	100.0	85.7	95.5	87.5	100.0	100.0	95.5
Platynini	3/2/4	100.0	100.0	100.0	100.0	―	―	―	―
Chlaeniini+Panagaeini+Oodini*	15/13/13	100.0	76.9	100.0	92.7	86.7	92.3	100.0	92.7
Lebiini+Cyclosomini	15/6/13	93.3	83.3	100.0	94.1	73.3	83.3	100.0	85.3

a Asterisks indicate taxa for which the homogeneity of the variance–covariance matrix of the data set was confirmed.

b L1, L2, and L3 indicate first, second, and third instar larvae, respectively.

Based on the results of the discriminant analyses, factors affecting discrimination accuracy were analyzed by constructing generalized linear models (GLMs). Whether each specimen was correctly classified to its own instar was considered a binary variable (0 for incorrectly classified, 1 for correctly classified), and logit-link functions with binomial error structures were applied in the GLMs. To examine differences in discrimination accuracy among taxa, the analysis was initially performed using the combined data set for the eight within-family taxa, with taxa and larval instar as explanatory variables. A best model was constructed from a full model (i.e., one containing all predictors, including their interactions) using the function stepAIC in the R package MASS; this function selects the most parsimonious model based on minimizing the Akaike information criterion (AIC) values. Subsequent analyses were performed for each within-family taxa, with larval instar as the only explanatory variable. All statistical analyses were conducted using R version 2.9.0 [27].

## Results

With data set 1, the LDA and MDA results were similar (Table 1; for coefficients of the classification functions, see Tables S2 and S3 in the Supporting Information). In both cases, the correct classification rate was 71% for overall Carabidae, but analyses for individual taxa produced higher discriminant accuracies. In particular, the discriminant accuracy was rather improved in Trechinae, Pterostichini, Sphodrini, Platynini, Chlaeniini+Panagaeini+Oodini, and Lebiini+Cyclosomini, with the values increasing by more than 10%. In contrast, the correct classification rate did not improve very much in Harpalini, which had the lowest value in both analyses. Values in Zabrini were intermediate between these two cases. Among the instars, in all but one case, the second instar had the lowest discriminant accuracy.

In data set 2, the results were not consistent between LDA and MDA, except that the discriminant accuracy was higher in the within-family taxa analyses than for overall Carabidae. Compared with data set 1, the correct classification rate was higher in most taxa, although in some taxa, it became lower; in particular, the value decreased by about 9% for Lebiini+Cyclosomini in the MDA. In contrast to data set 1, no conspicuous differences in discriminant accuracy were found among the larval instars.

The results of the GLMs (Table 2) were consistent with those of the discriminant analyses. In data set 1, the best models for overall Carabidae included taxa and larval instar as explanatory variables in both LDA and MDA. Larvae of Zabrini, Harpalini, and second-instar larvae were more frequently incorrectly classified than were other taxa or instars. Subsequent analyses with individual within-family taxa data sets showed that second-instar larvae had lower correct classification rates than did the other instar larvae for Harpalini in LDA [β (estimated parameter, mean value ± SE) = –0.79±0.56, z-value = –2.17, *P = *0.030] and for Zabrini (β = –1.44±0.67, z-value = –2.15, *P = *0.032) and Harpalini (β = –0.98±0.37, z-value = –2.68, *P = *0.0073) in MDA. In data set 2, the best model for overall Carabidae in LDA included no explanatory variables, indicating that discriminant accuracy did not differ among taxa or larval instars. The best model for MDA included larval instar as an explanatory variable, but an estimated parameter was only marginally significantly different from zero. The analyses of the individual within-family taxa data sets found differences in discriminant accuracy among larval instars, but only for Pterostichini in MDA, with second-instar larvae being more frequently incorrectly classified than the other instars (β = –1.67±0.75, z-value = –2.24, *P = *0.025).

**Table 2:** Results of GLMs that analyzed the effects of taxa and larval instar on the correct classification rates of linear (LDA) and Mahalanobis squared distance-based (MDA) discriminant analyses.

Factors^a^	LDA				MDA			
	Estimates	SE	z-value	*P*	Estimates	SE	z-value	*P*
Data set 1								
(Intercept)	2.67	0.61	4.36	<0.001	2.92	0.62	4.71	<0.001
Trechinae	–1.01	0.65	–1.55	0.121	–0.66	0.67	–1.00	0.319
Pterostichini	–0.73	0.63	–1.17	0.242	–0.70	0.63	–1.12	0.264
Zabrini	–1.41	0.65	–2.16	0.031	–1.29	0.66	–1.95	0.051
Harpalini	–1.62	0.62	–2.62	0.009	–1.70	0.62	–2.73	0.006
Sphodrini	–0.21	0.80	–0.27	0.791	–0.21	0.80	–0.26	0.796
Platynini	0.21	0.94	0.23	0.822	0.21	0.95	0.22	0.824
Lebiini+Cyclosomini	–0.06	0.79	–0.08	0.939	0.25	0.85	0.29	0.770
Second instar	–0.38	0.22	–1.74	0.081	–0.81	0.23	–3.56	<0.001
Third instar	0.33	0.24	1.36	0.173	0.12	0.26	0.46	0.643
Data set 2								
(Intercept)	1.91	0.16	12.19	<0.001	1.95	0.27	7.32	<0.001
Second instar	–	–	–	–	−0.37	0.40	−0.94	0.346
Third instar	–	–	–	–	0.91	0.50	1.82	0.068

a Model of each dataset was constructed from a full model (i.e., one containing all predictors, including the interactions) using the function stepAIC in the R package MASS.

## Discussion

The method presented here assumes that in insects, the size ratio between successive developmental stages is constant. This pattern has already been reported; one of the earliest and best-known examples was published by Dyar (1890) (cited in [1]), who showed that in Lepidoptera, larval head width increased in a regular linear progression in successive instars by a ratio (range: 1.3–1.7) that was constant for a given species (Dyar’s rule). This size relationship has also been reported for Carabidae [19]. However, importantly, these reports only included larval stages; larval and adult body sizes were not combined. This new method includes the adult stage together with larval stages and can be considered an extension of Dyar’s rule.

Discriminant analysis was performed using two analytical methods (LDA and MDA) with two types of data sets. In all four cases, discriminant accuracy was higher in data sets that were derived from related species compared with a data set for overall Carabidae. For the eight within-family taxa that were examined, in all cases, the correct classification rate exceeded 80%, and for five taxa, it was more than 90%. Given that no reliable method has been established to identify the instar of field-collected insect larvae, these values are deemed to have sufficient accuracy as an analytical method for field-collected samples. The chief advantage of this technique is that the instar can be identified even when only one specimen is available per species if a classification function is determined for the group to which the focal species belongs. This contrasts with previous procedures for identifying larval instars, which required large numbers of identified samples and subsequent labor-intensive assessments (i.e., constructing frequency histograms of measurements of a sclerotized body part and determining the instar of each sample; [1] Figs. 6.12).

The results were also used to identify factors that affected discrimination accuracy. The analysis of data set 1 for overall Carabidae showed that second-instar larvae had a lower correct classification rate compared to first- and third-instar larvae. This is likely because although the range of the second instar can overlap with the ranges of the first and third instars on the adult size–larval size plane, the ranges of the first and third instars can only overlap with the other instars near their upper and lower limits, respectively (Fig. 1). Moreover, in data set 1, Zabrini and Harpalini exhibited lower correct classification rates compared with other taxa. This is probably because in Carabidae, the diversification of larval head morphology is associated with feeding habit. Although larvae of most carabids are carnivorous, larvae of Zabrini and Harpalini diversify with respect to their food habits, ranging from carnivory to omnivory and granivory [28]. Granivorous larvae have larger heads with more robust mandibles, which are used to crush seeds, compared with carnivorous larvae [29], [30]. Therefore, the head width of a given larval instar among species with similar adult sizes is more variable in these tribes (Zabrini and Harpalini) than in other groups that only include carnivorous larvae. This pattern would result in lower discrimination accuracies in these two taxa. Importantly, in data set 2, similar differences in discriminant accuracy among larval instars or taxa were not detected in either LDA or MDA. This result could be attributed to the fact that larval cerci length, the character that was added as an additional explanatory variable, is not associated with larval feeding habit.

Two promising avenues exist for future research. The first involves further improvements of the methodology. In this study, larval head width, cerci length and adult body length were used as indices of larval and adult sizes, respectively, because only these morphological measurements were available for many carabid species. However, if additional morphological characters were used as explanatory variables, the resulting classification function could have a higher discriminant accuracy, as was demonstrated by the improvement in discriminant accuracy with data set 2 (three explanatory variables) relative to data set 1 (two explanatory variables). For example, as indices of larval size, additional body parts that are not associated with larval feeding habit could be useful (e.g., pronotum width). Discrimination accuracy could also be improved by using other morphological characters that are more suitable than the ones that were used in this study. For example, as indices of adult size, morphological characters that are directly associated with larval size may be more appropriate (e.g., egg size). Also, applications of this new method to other insect groups and investigations of suitable indices of larval and adult size for individual taxa are also important. By using the resulting classification functions, future studies in other insect groups could include both larval stages and adults. The second research avenue involves integrating this new technique into existing research on species biology and ecology. In Carabidae, species identification with molecular sequence data is possible [31], and this technique was successfully applied to identify field-collected larvae [32]. Using the classification functions obtained here, it is now possible to identify the instar of carabid larvae with high reliability. Consequently, we can expand life history studies of Carabidae, which have predominantly focused on the adult stage (particularly for population dynamics), to include larval instars. Because carabid beetles are widely used as environmental indicators [28], the availability of information on larval stages would be beneficial from the perspectives of both basic and applied biology.

## Supporting Information

Table S1 Species data
(XLS)

Table S2 Coefficients of discriminant functions of larvae in linear discriminant analyses
(PDF)

Table S3 Coefficients of discriminant functions† of larvae in Mahalanobis’s squired discriminant analyses
(PDF)
