# Supplementary material for: A Novel Technique for Identifying the Instar of Field-Collected Insect Larvae
Source: PLoS One. 2013 Feb 28;8(2):e57836. doi: 10.1371/journal.pone.0057836 (PMC3585218; doi:10.1371/journal.pone.0057836)
Supplement: Table S2 — Coefficients of discriminant functions of larvae in linear discriminant analyses (PDF) [file pone.0057836.s002.pdf]

Table S2. Coefficients of discriminant functions† of larvae in linear discriminant analyses.

	L1	L2	L3
Data set 1			
Carabidae overall			
LHW‡	66.84089	47.27164	23.70925
ABL‡	-57.2962	-48.1358	-37.3922
Constant	47.66821	38.87176	32.8045
Trechinae			
LHW	161.8291	102.642	16.93747
ABL	-80.1983	-59.0782	-28.7743
Constant	44.01691	29.45682	20.74781
Pterostichini			
LHW	241.2803	181.1821	112.5302
ABL	-184.846	-152.708	-116.13
Constant	161.8079	121.4834	89.54539
Zabrini			
LHW	324.3246	278.7008	210.1345
ABL	-293.724	-262.302	-216.746
Constant	231.459	191.4638	145.5916
Harpalini			
LHW	158.6042	131.745	99.47848
ABL	-136.632	-119.848	-99.614
Constant	101.1882	83.04846	65.49271
Sphodrini			
LHW	219.1408	142.9357	64.4097
ABL	-169.126	-134.69	-101.426
Constant	154.5559	115.88	96.741
Platynini			
LHW	568.741	429.1365	278.6586
ABL	-358.689	-292.205	-219.785
Constant	264.4045	191.6799	134.7316
Chlaeniini+Panagaeini+Oodini			
LHW	561.0628	482.3337	380.9409
ABL	-503.279	-449.807	-383.59
Constant	506.0521	416.5338	326.7197
Lebiini+Cyclosomini			
LHW	181.184	102.7227	-4.47083
ABL	-111.36	-85.7639	-48.9121
Constant	80.32016	64.30503	54.99904
Data set 2			
Carabidae overall			
LHW	60.73174	46.06669	24.90331
LCL‡	22.43293	15.00523	8.14633
ABL	-66.5849	-55.7111	-42.1643
Constant	55.93541	44.10725	34.64037
Trechinae			
LHW	216.6878	124.8939	17.21621
LCL	-44.9372	-12.6434	12.75612
ABL	-79.028	-56.4291	-25.722

Constant	41.54587	24.88869	13.72341
Pterostichini			
LHW	242.3043	182.0722	102.0951
LCL	-12.999	-4.9346	10.37876
ABL	-192.188	-160.19	-126.515
Constant	178.3012	131.7877	101.6968
Zabrini			
LHW	406.5001	376.9094	292.7943
LCL	83.70253	86.70236	36.12428
ABL	-373.106	-354.97	-274.245
Constant	276.3323	253.6322	164.5047
Harpalini			
LHW	247.4183	217.7776	177.9104
LCL	70.57263	49.8995	28.69645
ABL	-243.724	-213.723	-176.055
Constant	177.7505	141.8675	104.9906
Sphodrini			
LHW	318.9386	275.9005	221.5226
LCL	-26.5975	-61.8642	-76.7773
ABL	-328.386	-289.373	-252.802
Constant	349.9719	297.4761	260.4042
Platynini			
LHW	152.5703	63.55322	-96.1948
LCL	498.4324	448.9123	438.8477
ABL	-594.198	-500.197	-402.849
Constant	541.0639	399.8458	295.927
Chlaeniini+Panagaeini+Oodini			
LHW	585.8192	494.6164	386.8051
LCL	42.59197	37.9755	33.019
ABL	-552.355	-489.131	-418.245
Constant	547.8753	444.9459	353.1497
Lebiini+Cyclosomini			
LHW	196.244	116.5801	-8.76183
LCL	21.44957	-5.92959	-65.195
ABL	-145.999	-111.366	-58.4513
Constant	113.26	90.33355	96.02412

† Discriminant functions for instar *i* in full data set: $Y_i=A_i$*LHW+B_i*ABL+C_i (A_i, coefficient for LHW; B_i, coefficient for ABL; C_i, constant); and those in reduced data set: $Y_i=D_i$*LHW+E_i*LCLL+ F_i*ABL+G_i (D_i, coefficient for LHW; E_i, coefficient for LCL; F_i, coefficient for ABL; G_i, constant)

‡ LHW, ln(larval head width+1); ABL, ln(adult body length+1); LCL, ln(larval cerci length+1).

Table S3. Coefficients of discriminant functions† of larvae in Mahalanobis's squired discriminant analyses.

	L1	L2	L3
Data set 1			
Carabidae overall			
H	51.80625	81.97827	86.11453
I	-20.9505	-37.9039	-46.7709
J	13.51794	23.49591	31.79544
Trechinae			
H	1428.263	713.4264	242.9928
I	-332.109	-252.074	-123.216
J	84.69384	94.97588	67.85622
Pterostichini			
H	152.0406	188.6665	178.3857
I	-68.3898	-100.755	-109.728
J	42.0233	65.1482	79.20696
Zabrini			
H	153.6175	218.2888	221.6016
I	-96.9352	-136.026	-157.833
J	76.7133	109.9042	128.3866
Harpalini			
H	104.5001	121.5033	123.6487
I	-57.5869	-75.1423	-88.4746
J	39.65218	54.83496	72.122
Sphodrini			
H	300.8487	287.5374	170.3571
I	-99.9505	-125.973	-99.2476
J	43.70132	73.95332	76.22642
Platynini			
H	694.3434	719.4734	668.011
I	-231.686	-339.537	-408.445
J	97.38442	179.0401	270.2256
Chlaeniini+Panagaeini+Oodini			
H	343.999	229.9661	236.8191
I	-193.016	-153.877	-171.602
J	130.0445	138.6507	149.1981
Lebiini+Cyclosomini			
H	488.0914	343.9881	256.4319
I	-128.243	-128.83	-108.928
J	43.46184	61.28817	59.02891
Data set 2			
Carabidae overall			
K	45.42648	64.96717	78.31769
L	-6.26258	4.753325	4.78928
M	-17.271	-33.6064	-47.2706
N	29.27815	21.99802	14.38887
O	-8.78706	-12.583	-10.658
P	15.95592	27.74663	39.56977
Trechinae			
K	5254.256	5938.251	668.2006

L	-2313.66	-1959.95	-260.336
M	-753.764	-1369.37	-191.722
N	1417.271	904.4048	215.1857
O	222.9146	368.7196	7.428916
P	142.9288	346.2765	97.91833
Pterostichini			
K	359.3947	409.8373	168.1202
L	-115.791	-139.964	-48.591
M	-105.266	-128.436	-70.7476
N	55.83695	83.80718	54.62191
O	21.09488	19.79396	-14.1127
P	51.76379	69.68613	71.42169
Harpalini			
K	127.7473	168.3239	276.0638
L	-53.1856	1.027514	-26.0834
M	-59.7792	-116.659	-196.747
N	216.5118	78.57102	68.82532
O	-53.1187	-33.679	-19.197
P	67.46472	103.065	171.1412
Sphodrini			
K	510.2529	253.526	129.9539
L	-367.819	-28.3853	-46.0487
M	-125.634	-158.213	-68.3365
N	453.0543	55.24396	151.3579
O	53.95735	3.760522	-47.2471
P	69.21127	135.8993	97.12512
Chlaeniini+Panagaeini+Oodini			
K	371.864	232.1164	296.0656
L	20.0607	6.433654	-4.4949
M	-230.719	-162.908	-179.273
N	14.44218	7.008834	5.643864
O	-27.1432	-12.2351	-3.60737
P	181.0585	156.0078	148.9206
Lebiini+Cyclosomini			
K	512.9713	167.6365	470.1958
L	-177.144	-2.17682	115.7219
M	-115.28	-68.0207	-264.689
N	314.4962	139.8031	63.60029
O	-7.51098	-27.1939	-57.643
P	49.27814	39.66429	201.8818

†Discriminant functions for instar *i* in full data set: $Y_i = (\mathrm{LHW}\ \mathrm{ABL}) \begin{pmatrix} H_i & I_i \\ I_i & J_i \end{pmatrix} \begin{pmatrix} \mathrm{LHW} \\ \mathrm{ABL} \end{pmatrix}$; and

those in reduced data set: $Y_i = (\mathrm{LHW}\ \mathrm{LCL}\ \mathrm{ABL}) \begin{pmatrix} Ki & Li & Mi \\ Li & Ni & Oi \\ Mi & Oi & Pi \end{pmatrix} \begin{pmatrix} \mathrm{LHW} \\ \mathrm{LCL} \\ \mathrm{ABL} \end{pmatrix}$
