# Supplementary material for: A Novel Technique for Identifying the Instar of Field-Collected Insect Larvae
Source: PLoS One. 2013 Feb 28;8(2):e57836. doi: 10.1371/journal.pone.0057836 (PMC3585218; doi:10.1371/journal.pone.0057836)
Supplement: Table S3 — Coefficients of discriminant functions† of larvae in Mahalanobis’s squired discriminant analyses (PDF) [file pone.0057836.s003.pdf]

Table S3. Coefficients of discriminant functions† of larvae in Mahalanobis's squired discriminant analyses.

	L1	L2	L3
Data set 1			
Carabidae overall			
H	51.80625	81.97827	86.11453
I	-20.9505	-37.9039	-46.7709
J	13.51794	23.49591	31.79544
Trechinae			
H	1428.263	713.4264	242.9928
I	-332.109	-252.074	-123.216
J	84.69384	94.97588	67.85622
Pterostichini			
H	152.0406	188.6665	178.3857
I	-68.3898	-100.755	-109.728
J	42.0233	65.1482	79.20696
Zabrini			
H	153.6175	218.2888	221.6016
I	-96.9352	-136.026	-157.833
J	76.7133	109.9042	128.3866
Harpalini			
H	104.5001	121.5033	123.6487
I	-57.5869	-75.1423	-88.4746
J	39.65218	54.83496	72.122
Sphodrini			
H	300.8487	287.5374	170.3571
I	-99.9505	-125.973	-99.2476
J	43.70132	73.95332	76.22642
Platynini			
H	694.3434	719.4734	668.011
I	-231.686	-339.537	-408.445
J	97.38442	179.0401	270.2256
Chlaeniini+Panagaeini+Oodini			
H	343.999	229.9661	236.8191
I	-193.016	-153.877	-171.602
J	130.0445	138.6507	149.1981
Lebiini+Cyclosomini			
H	488.0914	343.9881	256.4319
I	-128.243	-128.83	-108.928
J	43.46184	61.28817	59.02891
Data set 2			
Carabidae overall			
K	45.42648	64.96717	78.31769
L	-6.26258	4.753325	4.78928
M	-17.271	-33.6064	-47.2706
N	29.27815	21.99802	14.38887
O	-8.78706	-12.583	-10.658
P	15.95592	27.74663	39.56977
Trechinae			
K	5254.256	5938.251	668.2006

L	-2313.66	-1959.95	-260.336
M	-753.764	-1369.37	-191.722
N	1417.271	904.4048	215.1857
O	222.9146	368.7196	7.428916
P	142.9288	346.2765	97.91833
Pterostichini			
K	359.3947	409.8373	168.1202
L	-115.791	-139.964	-48.591
M	-105.266	-128.436	-70.7476
N	55.83695	83.80718	54.62191
O	21.09488	19.79396	-14.1127
P	51.76379	69.68613	71.42169
Harpalini			
K	127.7473	168.3239	276.0638
L	-53.1856	1.027514	-26.0834
M	-59.7792	-116.659	-196.747
N	216.5118	78.57102	68.82532
O	-53.1187	-33.679	-19.197
P	67.46472	103.065	171.1412
Sphodrini			
K	510.2529	253.526	129.9539
L	-367.819	-28.3853	-46.0487
M	-125.634	-158.213	-68.3365
N	453.0543	55.24396	151.3579
O	53.95735	3.760522	-47.2471
P	69.21127	135.8993	97.12512
Chlaeniini+Panagaeini+Oodini			
K	371.864	232.1164	296.0656
L	20.0607	6.433654	-4.4949
M	-230.719	-162.908	-179.273
N	14.44218	7.008834	5.643864
O	-27.1432	-12.2351	-3.60737
P	181.0585	156.0078	148.9206
Lebiini+Cyclosomini			
K	512.9713	167.6365	470.1958
L	-177.144	-2.17682	115.7219
M	-115.28	-68.0207	-264.689
N	314.4962	139.8031	63.60029
O	-7.51098	-27.1939	-57.643
P	49.27814	39.66429	201.8818

†Discriminant functions for instar *i* in data set 1: $Y_i = \begin{pmatrix}\mathrm{LHW} & \mathrm{ABL}\end{pmatrix}\begin{pmatrix}H_i & I_i \\ I_i & J_i\end{pmatrix}\begin{pmatrix}\mathrm{LHW} \\ \mathrm{ABL}\end{pmatrix}$; and those in data set 2: $Y_i = \begin{pmatrix}\mathrm{LHW} & \mathrm{LCL} & \mathrm{ABL}\end{pmatrix}\begin{pmatrix}Ki & Li & Mi \\ Li & Ni & Oi \\ Mi & Oi & Pi\end{pmatrix}\begin{pmatrix}\mathrm{LHW} \\ \mathrm{LCL} \\ \mathrm{ABL}\end{pmatrix}$, where LHW, ABL, and LCL are ln(larval head width+1), ln(adult body length+1), and ln(larval cerci length+1), respectively.
